# Transcriptomics analysis reveals the signal transduction mechanism of brassinolides in tea leaves and its regulation on the growth and development of *Camellia sinensis*

**DOI:** 10.1186/s12864-021-08179-9

**Published:** 2022-01-06

**Authors:** Qifang Jin, Zhong Wang, Yanni Chen, Yiping Luo, Na Tian, Zhonghua Liu, Jianan Huang, Shuoqian Liu

**Affiliations:** 1 Key Laboratory of Tea Science of Ministry of Education, Hunan Agricultural University, Changsha, China; 2 National Research Center of Engineering and Technology for Utilization of Botanical Functional Ingredients, Hunan Agricultural University, Changsha, China; 3 Co-Innovation Center of Education Ministry for Utilization of Botanical Functional Ingredients, Hunan Agricultural University, Changsha, China

**Keywords:** Brassinosteroids, Tea plant, Tea leaf, Growth and development, Signal transduction

## Abstract

**Background:**

Brassinosteroids (BRs) are a type of sterol plant hormone that play an important role in various biochemical and physiological reactions such as promoting cell growth, increasing biomass, and improving stress resistance.

**Results:**

To investigate the regulatory and molecular mechanism of BRs on the growth and development of tea plants (*Camellia sinensis* L.), changes in cell structure and gene expression levels of tea leaves treated with exogenous BRs were analyzed by electron microscopy and high-throughput Illumina RNA-Seq technology. The results showed that the number of starch granules in the chloroplasts and lipid globules increased and thylakoids expanded after BR treatment compared with the control. Transcriptome analysis showed that in the four BR treatments (CAA: BR treatment for 3 h, CAB: BR treatment for 9 h, CAC: BR treatment for 24 h, and CAD: BR treatment for 48 h), 3861 (1867 upregulated and 1994 downregulated), 5030 (2461 upregulated and 2569 downregulated), 1626 (815 upregulated and 811 downregulated), and 2050 (1004 upregulated and 1046 downregulated) differentially expressed genes were detected, respectively, compared with CAK (BR treatment for 0 h). Using Gene Ontology (GO) and Kyoto Encyclopedia of Genes and Genomes (KEGG) databases, metabolic pathway enrichment analysis showed that the differentially expressed genes of CAA vs. CAK, CAB vs. CAK, CAC vs. CAK, and CAD vs. CAK significantly enriched the functional categories of signal transduction, cell cycle regulation, and starch, sucrose, and flavonoid biosynthesis and metabolism pathways. We also found that after spraying BR, the key genes for caffeine synthesis were downregulated. The results of qRT-PCR coincided with the findings of transcriptomic analysis.

**Conclusions:**

The present study improved our understanding of the effects of BRs on the growth and development of tea leaves and laid the foundation for the in-depth analysis of signal transduction pathways of BRs in tea leaves.

**Supplementary Information:**

The online version contains supplementary material available at 10.1186/s12864-021-08179-9.

## Introduction

Brassinosteroids (BRs), known as the sixth category of plant hormones [1], are involved in various physiological and biochemical reactions, particularly plant growth and development by promoting cell growth, increasing biomass, and improving stress resistance [2]. A previous study revealed that spraying rice seedlings with BRs improved fresh weight by 22% and dry weight by 31.5% [3]. The application of BRs on cucumber leaves increased total soluble sugar, sucrose, hexose, and starch content, followed by enhancement of sugar metabolism activities involving sucrose phosphate synthase, sucrose synthase, and invertase [4]. In addition, studies have shown that exogenous spraying of BRs induces anthocyanin accumulation in *Arabidopsis thaliana* seedlings [5].

BRs also improve the survival rate and vitality of plants in adverse environments, which is of practical value to agricultural production [6]. Under low temperature, drought, and saline-alkali stress, BRs act as buffer to stress conditions by regulating the intracellular physiological environment, promoting normal physiological and biochemical metabolism, and enhancing plant stress resistance [7]. In rice seedlings grown under the conditions of low temperature, low sunlight, and high precipitation, when the roots were soaked in 0.01-mg/L BR solution, plant height, leaf number, leaf area, millet number, and root number, survival rate, and aboveground dry weight were higher than the control group [8]. In addition, BRs prevented chilling injuries in maize seedlings during germination and early growth stages, as well as reduced the yellowed maize leaf area, especially under the conditions of low temperature and low sunlight [9].

Cell expansion modifies the cell wall. Xyloglucan endoglycosyltransferase is a cell wall-modifying protein that adds new xylan during cell wall formation [10]. Studies have shown that the promotion of cell extension by BRs largely relies on the expression of the xyloglucan endoglycosyltransferase (*XET*) gene [11]. BR application to soybean hypocotyls increases cell wall plasticity, gene transcription, and BR activity during the early stage of cell elongation [12]. Similarly, the protein encoded by the loua (*TCH*) gene promotes the activity of *XET* enzymes in *Arabidopsis thaliana*, and its expression increases with BR treatment [13]. In *A. thaliana* mutants such as *det*, *cwf4*, and *cpd*, *TCH4* gene expression is downregulated, resulting in dwarf mutants [14]. The underlying mechanism of BRs involves relaxing the cell wall and promoting growth by regulating the expression of the *TCH4* gene [15]. Thus, BRs influence cell elongation by regulating the expression of cell elongation-related genes. BRs promote plant growth by increasing cell volume and promoting cell division [16]. BRs also upregulate cyclin (*CycD3*) gene transcription in a suspension cell culture of mutant *det2*. In general, *CycD3* is activated by cytokinins to promote cell division, indicating that BRs also promote cell division by activating *CycD3*.

The signal transduction pathway of BRs has been established and can be summarized into three steps [17]: (1) the perception and reception of a BR signal on the cell surface or plasma membrane; (2) the transmission of the BR signal in the cytoplasm; and (3) the amplification of the signal in the nucleus.

When the concentration of BRs in the cell is low or in the absence of BRs, BRI1 kinase inhibitor 1 (BKI1) located on the cell membrane binds to brassinosteroid insensitive 1 (BRI1) [18]. The functional deletion of the *OsBRI1* gene in rice results in dwarfing, shortened internode length, and smaller leaves [19]. The binding of BKI1 and BRI1 inhibits the interaction of BRI1 with co-receptor kinase BRI1-associated receptor kinase1 (BAK1), thus inhibiting the function of BRI1; meanwhile, Brassinosteroidinsensitive 2 (BIN2), a negative regulator of BR signal transduction, is activated and phosphorylates Brassinazole resistant 1 (BZR1) and BRI1 ems suppressor 1 (BES1), key transcription factors of the BR signaling pathway. Phosphorylated BZR1 and BES1 readily bond with the 14-3-3 protein and remain in the cytoplasm, losing its ability to bind to the target gene promoter in the nucleus [20]. However, phosphorylated BZR1 and BES1 are less stable and are easily degraded by proteasomes.

When the cellular concentration of BRs is high, BRs bind to the extracellular domain of BRI1 and promote the dissociation of BKI1 from BRI1 [21]. Moreover, BRI1 can better bind and activate downstream protein kinase BAK1 and activate downstream protein BR Signaling kinases (BSK) and constitutive differential growth 1 (CDG1), after which BSK1/CDG1 phosphorylates BRI1 suppressor 1 (BSU1), followed by BSU1 dephosphorylation of BIN2 to inactivate BIN2, resulting in the dephosphorylation of downstream transcription factors BZR1 and BES1 [22]. Dephosphorylated BZR1 and BES1 are transferred to and accumulate in the nucleus, and the DNA binding ability of downstream target genes is enhanced, which can directly regulate the expression of related genes downstream of the BR signal pathway and amplify the signal step-by-step, inducing a series of physiological and biochemical reactions, thus regulating plant growth and development [23].

To date, the effects of exogenous BR spraying on the growth and development of *Arabidopsis thaliana* and rice have been studied, and the BR signal pathway in model plants has also been investigated [24]. Exogenous spraying of BRs on tea leaves enhanced plant defense against *colletotrichum gloeosporioides* by activating phenylpropanoid pathway in *C. sinensis* [25]. Meanwhile, exogenous 24-epibrassinolide (EBR, a bioactive BR) sharply increased PAL activity of *C. gloeosporioides* inoculated tea leaves. Analysis of genes expression involved in phenylpropanoid pathway showed that both exogenous EBR treatment and *C. gloeosporioides* inoculation increased transcript levels of phenylalanine ammonia-lyase (*CsPAL*), cinnamate 4-hydroxylase (*CsC4H*), and 4-coumarate–CoA ligase (*Cs4CL*). Besides, exogenous BRs increased the contents of catechins and theanine increased though no significant effect was observed on caffeine [26], which provided a novel way to regulate tea quantity. Li and his collaboratories reported that BR enhanced flavonoid level in tea leaves by inducing an increase in the endogenous concentration of nitric oxide (NO) [27]. Recently, it was reported that exogenous BRs improved theanine level in tea leaves under sub high temperature by regulating the activity of enzymes and genes involved in theanine biosynthesis [28]. Above researches suggest that BRs play an important role on the quantity of tea leaves and physiology of tea plant. However, the transduction and action mechanism of BR in tea leaves are still unclear.

In the present work, the size of starch grains, the number of lipid globules, and the size of thylakoids in the chloroplasts of different samples treated with BRs at different time points were assessed by electron microscopy. Differentially expressed genes (DEGs) related to BR signal transduction, cell division, starch synthesis, flavonoid biosynthesis, and sugar synthesis were qualitatively and quantitatively analyzed by high-throughput Illumina RNA-Seq, laying the foundation for further analysis of the effects of exogenous BR spraying on the growth and development of tea leaves and elucidation of the BR signal transduction pathway in tea leaves.

## Materials and methods

### Experiment material

“Bixiangzao” tea plants were planted in a greenhouse at a temperature of 26.0 ± 3.0 °C and relative humidity of 86.0% ± 3.0%. The same concentration (0.005 mol/L) of BRs was sprayed on tea plants (first-leaf position) in the same growth environment. The spray solution was prepared as follows: 100 mL water + 10 μL BR (0.005 mol/L). There were five treatment groups, in which BRs were sprayed for 0 h, 3 h, 9 h, 24 h, and 48 h (CAK, CAA, CAB, CAC, and CAD, respectively). There were three biological replicates for each set. Samples were wrapped in tinfoil paper and stored in an ultra-low − 80 °C freezer at − 80 °C after solidification in liquid nitrogen. In addition, fresh tea leaves from different processed samples were collected and placed in a fixing solution (Servi Biotechnology Co., Ltd.) assessment by electron microscopy.

### Observation of cell ultrastructure by transmission electron microscope

The leaf tissues of tea plants (first-leaf position) of different treatments were cut into small pieces with dimensions of 1 mm × 1 mm. After fixation, dehydration, embedding, sectioning, and double-staining with uranium acetate and lead citrate, the ultrastructure of the cells was observed using a Hitachi Hmur7650 transmission electron microscope [Hitachi (China) Co., Ltd.].

### RNA extraction and detection

RNA was extracted from tissues using the Tiangen polysaccharide and polyphenol kit, following strict quality control protocols. The quality control method was mainly conducted using the Agilent 2100 Bioanalyzer to accurately assess RNA integrity.

### Library construction and quality inspection

mRNA was obtained by removing ribosomal RNA from the extracted total RNA. Subsequently, the mRNAs were randomly interrupted with divalent cations in the NEB fragmentation buffer, and a library was constructed according to the NEB normal library building method. The NEB general library construction was performed as follows: using fragmented mRNA as a template and random oligonucleotides as primers, the first cDNA strand was synthesized in the M-MuLV reverse transcriptase system. Then, RNaseH was used to degrade the RNA strand and also used in the DNA polymerase I system. Next, the second strand of cDNA was synthesized using dNTPs as raw materials. The purified double-stranded cDNA underwent end-repair and the addition of polyA tails and sequencing adapters. The 250- to 300-bp cDNA was screened with AMPure XP beads, PCR amplification was performed, and the PCR product was purified again with AMPure XP beads to obtain a library. The kit used for library construction was the NEBNext® Ultra™ RNA Library Prep Kit (Illumina®) [Gene Biotechnology International Trade (Shanghai) Co., Ltd.].

After the library was constructed, the Qubit 2.0 Fluorometer (Shanghai Hengfei Biological Technology Co., Ltd.) was used for preliminary quantification, the library was diluted to 1.5 ng/μL, and the Agilent 2100 Bioanalyzer [Agilent Technologies (China) Co., Ltd.] was then used to detect the insert size of the library. After the insert size met the expectation, qRT-PCR was used to measure the effective concentration of the library. Accurate quantification (the effective concentration of the library > 2 nmol/L) ensured the quality of the library.

### Transcriptome sequencing and alignment

The library was constructed on the Illumina sequencer for paired-end sequencing to obtain raw reads. Quality control was performed through SeqPrep (Lexogen Biotechnology, Vienna, Austria) software to obtain high-quality control data (clean reads), and the Q20, Q30, and GC content (GC) and sequence repetition level of clean reads were calculated. After comparing the clean reads to the reference genome using HISAT2 software, these were assembled by Cufflinks software to obtain the difference information between this sequencing and the original annotations. Finally, FPKM was used to calculate gene expression levels.

### DEGs and enrichment analysis

The DEGs were calculated and screened by DESeq2 software and were defined as: |log2^FoldChange^| > 2, P-adjust < 0.05, where fold change represents the ratio of expression levels between two samples (groups). ClusterProfile software was used to perform GO and KEGG function enrichment analyses of DEGs. When the corrected *P* value (P-adjust) was < 0.05, the GO function and the KEGG pathway functions were considered significantly enriched, and the Tbtools software (the developer is Dr. Chen Chengjie from South China Agricultural University) was used to construct figures.

### Transcriptome data verification

Twelve DEGs were randomly selected for expression level verification (Table 1). The RNAprep Pure Plant Kit [Tiangen Biochemical Technology (Beijing) Co., Ltd.] was used to extract total RNA, and the Fastking gDNA DispelllingRT SuperMix kit [Tiangen Biochemical Technology (Beijing) Co., Ltd.] was used to synthesize cDNA as a real-time fluorescent quantitative PCR template, using three biological replicates. Using CsGAPDH (GE651107) as the internal reference gene, the Applied Biosystems fluorescence quantitative PCR instrument was used to perform qRT-PCR. The reaction system was based on the protocol provided in the Transstart® Tip Green qPCR superMix kit (Beijing Quanshijin Biotechnology Co., Ltd.). The reaction procedure was as follows: 94 °C for 30 s; followed by 40 cycles of 94 °C for 5 s, 60 °C for 30 s. The 2-ΔΔCt method was used to calculate gene expression levels.

**Table 1:** Primer sequences

Gene ID	Gene Name	Forward primer sequence (5′-3′)	Reverse primer sequence (5′-3′)
CSS0040899	BAK1	CGACCAGCGGTACAATCCAT	CAGTGTTGGTGTACTCGGGG
CSS0017722	BES1	TGGTGGGTCAGCTTCAGCAA	ATGGCATTGGCAGCGTAACG
CSS0043647	BSU1	TTCGCATGATAGCAGCCAGT	CAAACTTGCCCACACACTCG
CSS0024623	SPS	GATGTTCTCGGGGATGCGAT	GGAATCACGACCAAGCTCCA
CSS0015657	SBE	TTGTGCAAGAGAGGGCCATT	GCTCCTCAACGGTAACACCT
CSS0033593	POR	GCCACGACAGGTTTGTTCAG	CAACCTGTGCAAGTCGCTTT
CSS0030876	DFR	CCCTTGCAGCACAATTCCCAGAG	GAATCGGCTATGCTCCTCACACTG
CSS0039817	CycD3	AGCTGCGATACCTCGAACG	GGTGCCAATCTCATCTGCTG
CSS0008835	TS	TCTCAACAATGGCGGCTGCTTAC	TGGAGGAGGTGGAGGATTTGATG
CSS0034978	GS	GCAAACGCCACAAGAACGAATACG	ACTTCAAGGACACGACCATCAACC
CSS0028985	ACD	CAGATACCCACAACCACCTTGCTAG	TCCGCCACCAATATCAATGACTTC
CSS0001813	CBF	GTTCCCGAATAGCCGAGTCA	TGGAGGAAGAGATCGGTGGA
CsGAPDH	GAPDH	TTGGCATCGTTGAGGGTCT	CAGTGGGAACACGGAAAGC

### Statistical analysis

Microsoft Excel 2016, SPSS 17.0, and MeV 4.9.0 were used for statistical analysis. The significant difference was analyzed by single-factor ANOVA (*P* < 0.05).

## Results

### Ultrastructure of leaf cells

**Fig. 1:**
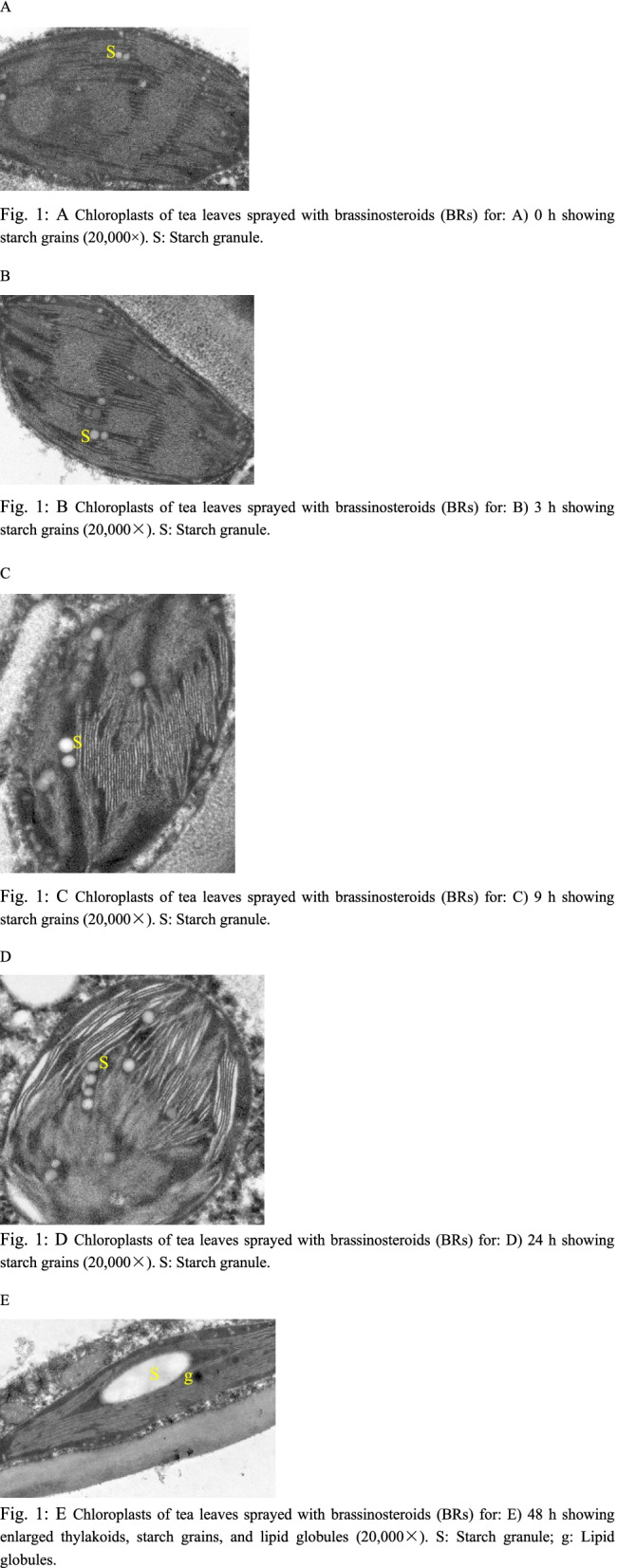
**A** Chloroplasts of tea leaves sprayed with brassinosteroids (BRs) for: **A**) 0 h showing starch grains (20,000×). s: Starch granule. **B** Chloroplasts of tea leaves sprayed with brassinosteroids (BRs) for: **B**) 3 h showing starch grains (20,000×). s: Starch granule. **C** Chloroplasts of tea leaves sprayed with brassinosteroids (BRs) for: **C**) 9 h showing starch grains (20,000×). s: Starch granule. **D** Chloroplasts of tea leaves sprayed with brassinosteroids (BRs) for: **D**) 24 h showing starch grains (20,000×). s: Starch granule. **E** Chloroplasts of tea leaves sprayed with brassinosteroids (BRs) for: **E**) 48 h showing enlarged thylakoids, starch grains, and lipid globules (20,000×). s: Starch granule; g: Lipid globules

Electron microscopic observation showed that among the five treatments studied, the largest starch grains were found in the samples sprayed with BRs for 48 h, with lipid globules in the chloroplast (Fig. 1: E). There were a few starch grains in the chloroplast of tea leaves sprayed with BRs for 0 h. The chloroplasts of tea leaves sprayed with BRs for 3 h and 9 h showed minimal cellular changes, and the starch grains were approximately round in shape (Fig. 1: B–C). After spraying BRs for 24 h, the number of starch grains began to increase significantly, and the starch grains were round and arranged in order. In the chloroplast of tea leaves sprayed with BRs for 48 h, the starch grains were long and oval in shape (Fig. 1: E). In the chloroplasts of the five tea plants studied, all starch grains were distributed along the long axis of the chloroplast, and the electron density of starch grains was lower (Fig. 1: A–E). In addition, lipid globules were also found in the chloroplasts of the five treated tea trees (Fig. 1: E). In chloroplasts with a large number of lipid globules, thylakoids were enlarged (Fig. 1: E). With increasing BR spraying time, the starch grains in tea leaves became larger.

### Global expression profile analysis of tea leaves

The samples of fresh tea leaves treated with CAK (0 h after BR treatment) and different BR treatment durations (CAA, CAB, CAC, and CAD) were analyzed by RNA-Seq, and three independent repeats were conducted. The average clean reads were 6.89 Gb in length (Table 2), and GC percentages ranged from 43.12 to 44.21%. The base percentage of Q30 ranged from 90.53 to 94.18%, indicating that the data obtained by transcriptome sequencing was of high quality.

**Table 2:** Quality statistics of reads after filtering

Sample	Total raw reads (Mb)	Total clean reads (Mb)	Total clean bases (Gb)	Clean reads Q20 (%)	Clean reads Q30 (%)	Clean reads ratio (%)	GC percent (%)
CAA1	47.76	46.76	7.01G	97.83	93.95	97.91	43.86
CAA2	47.33	46.41	6.96G	97.88	94.06	98.06	43.95
CAA3	47.38	46.46	6.97G	97.87	94.06	98.06	44.01
CAB1	46.68	45.90	6.89G	97.90	94.09	98.33	43.19
CAB2	45.95	45.10	6.76G	97.93	94.15	98.15	43.57
CAB3	46.86	45.99	6.90G	97.76	93.80	98.14	43.12
CAC1	50.39	49.32	7.40G	97.13	92.46	97.88	43.50
CAC2	48.64	47.79	7.17G	97.94	94.18	98.25	44.21
CAC3	47.35	46.33	6.95G	97.81	93.92	97.85	43.75
CAK1	45.74	44.63	6.69G	97.92	94.13	97.57	43.83
CAK2	44.85	43.83	6.57G	97.77	93.80	97.73	43.91
CAK3	46.76	45.85	6.88G	97.87	93.99	98.05	43.78

**Fig. 2:**
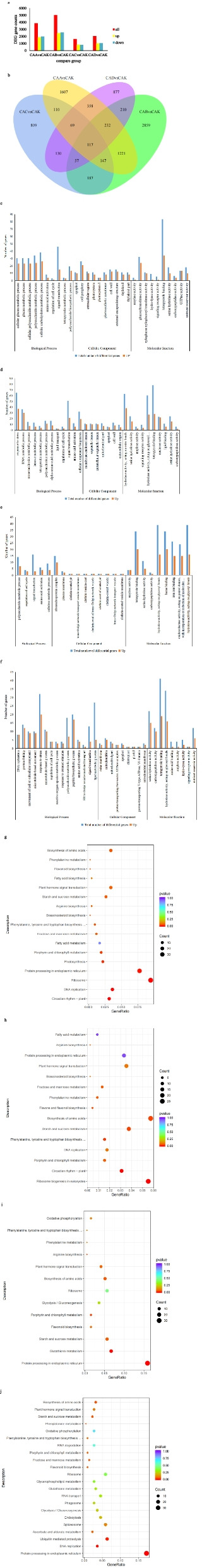
**a** The number of differential genes up- or downregulated by the four comparison combinations (CAA vs. CAK, CAB vs. CAK, CAC vs. CAK, and CAD vs. CAK). **b** Venn diagram of four comparative combinations. **c–f** Column chart of GO enrichment analysis of upregulated differentially expressed genes in **c** CAA vs. CAK, **d** CAB vs. CAK, **e** CAC vs. CAK, and **f** CAD vs. CAK. **g**–**j**, **g** CAA vs. CAK upregulation in the bubble map of differentially expressed genes by KEGG enrichment analysis. KEGG enrichment analysis bubble chart of upregulated genes in **h** CAB vs. CAK, **i** CAC vs. CAK, and **j** KEG CAD vs. CAK

On the basis of measuring the gene expression level of each sample, a DEGseq algorithm was used to analyze the DEGs in fresh tea leaves treated with CAK (BRs for 0 h) and BRs for different durations (CAA, CAB, CAC, and CAD). The results showed that compared with CAK (0 h BR treatment), CAA (spraying BR 3 h) had 1867 genes upregulated and 1994 genes downregulated. CAB (spraying BR for 9 h) had 2461 genes upregulated and 2569 genes downregulated. CAC (spraying BR for 24 h) had 815 genes upregulated and 811 genes downregulated. A total of 1004 genes were upregulated and 1046 were downregulated when BRs were sprayed for 48 h (CAC) compared with the 0-h BR treatment (CAK) (Fig. 2a). As can be seen from the Wayne diagram (Fig. 2b), there were 117 DEGs were shared among all groups. Compared with CAK, upregulated and downregulated genes accounted for almost half of the four groups of treated samples. This may be due to the rapid stimulation of the expression of some genes after the exogenous spraying of BRs and the consumption of some genes involved in the tissue activities of tea leaves, resulting in the downregulation of expression. Among these, the total number of DEGs was the highest in CAB (the sample sprayed with BR for 9 h). The overall trend was that after exogenous BR spraying, the total number of DEGs initially increased and then sharply decreased. These included significantly upregulated genes that were related to BR signal transduction, cell division, and starch, sugar, and flavonoid metabolism such as starch-branching enzyme (BES), Cyc, granule-bound starch synthase (GBSS), sucrose phosphate synthase (SPS), dihydroflavonol− 4-reductase (DFR), leucoanthocyanidin reductase (LAR), anthocyanidin reductase (ANR), and UDP flavonoid glucosyl transferase (UFGT).

Based on GO taxonomic analysis, the functional enrichment of four comparative combinations (CAA vs. CAK, CAB vs. CAK, CAC vs. CAK, and CAD vs. CAK) was revealed (Fig. 2:c–f). Compared with CAK, the DEGs upregulated in CAA mainly enriched 30 subgroups in the functional categories of biological process, cell composition, and molecular function, including the polysaccharide metabolism process (GO:0005976), amino acid activation (GO:0043038), regulation of cell cycle (GO:0051726), signal transduction (GO:0007165), cell periphery (GO:0071944), amylase activity (GO:0016160), signal receptor activity (GO:0038023), tetrapyrrole binding (GO: 0046906), carboxypeptidase activity (GO: 0004180), and serine hydrolase activity (GO: 0017171). Compared with CAK, the DEGs upregulated in CAB mainly enriched 29 subgroups in the functional categories of biological process, cell composition, and molecular function, including the polysaccharide metabolism process (GO:0005976), tetrapyrrole metabolism process (GO:0033013), regulation of cell cycle (GO:0051726), signal transduction (GO:0007165), amino acid activation (GO:0043038), organelle lumen (GO:0043233), amylase activity (GO:0016160), signal receptor activity (GO:0038023), tetrapyrrole binding (GO:0046906), carboxypeptidase activity (GO: 0004180), and serine hydrolase activity (GO: 0017171). Compared with CAK, the DEGs upregulated in CAC mainly enriched 24 subgroups in the functional categories of biological process and molecular function, including the process of polysaccharide metabolism (GO:0005976), regulation of cell cycle (GO:0051726), signal transduction (GO:0007165), amino acid activation (GO:0043038), plasma membrane (GO:0005886), amylase activity (GO:0016160), tetrapyrrole binding (GO:0046906), carboxypeptidase activity (GO:0004180), and serine hydrolase activity (GO: 0017171). Compared with CAK, the upregulated DEGs in CAD mainly enriched 33 subgroups in the functional categories of biological process, cell composition, and molecular function, including the polysaccharide metabolism process (GO:0005976), amino acid activation (GO:0043038), signal transduction (GO:0007165), regulation of cell cycle (GO:0051726), mitochondria (GO: 0005739), tetrapyrrole binding (GO:0046906), carboxypeptidase activity (GO: 0004180), serine hydrolase activity (GO: 0017171), amino acid binding (GO:0016597), amylase activity (GO:0016160), and signal receptor activity (GO:0038023). It was observed that after BR spraying, DEGs related to polysaccharide metabolism, signal transduction, amino acids, tetrapyrrole binding, carboxypeptidase activity, amylase activity, and cell cycle regulation were upregulated. Carboxypeptidase can hydrolyze polypeptides into amino acids. Chlorophyll belongs to the category of tetrapyrrole derivatives. Enrichment analysis of KEGG metabolic pathways (Fig. 2: g–j) revealed that after BR spraying, the expression of protein processing-related genes in the endoplasmic reticulum was significantly upregulated. Protein processing in the endoplasmic reticulum includes glycosylation, hydroxylation, acylation, and disulfide bond formation, of which the most important is glycosylation. Almost all proteins synthesized in the endoplasmic reticulum are finally glycosylated. Genes related to starch and sucrose metabolism were significantly upregulated in CAC (BR spraying for 24 h). Genes related to ubiquitin-mediated proteolysis were significantly upregulated in CAD (BRs sprayed for 48 h). Ubiquitin-mediated proteolysis produces amino acids. GO and KEGG enrichment analyses showed that after spraying BRs onto tea leaves, genes related to sugar, starch, chlorophyll metabolism, the cell cycle, signal transduction, and amino acid synthesis were upregulated.

### qRT-PCR analysis of DEGs

**Fig. 3:**
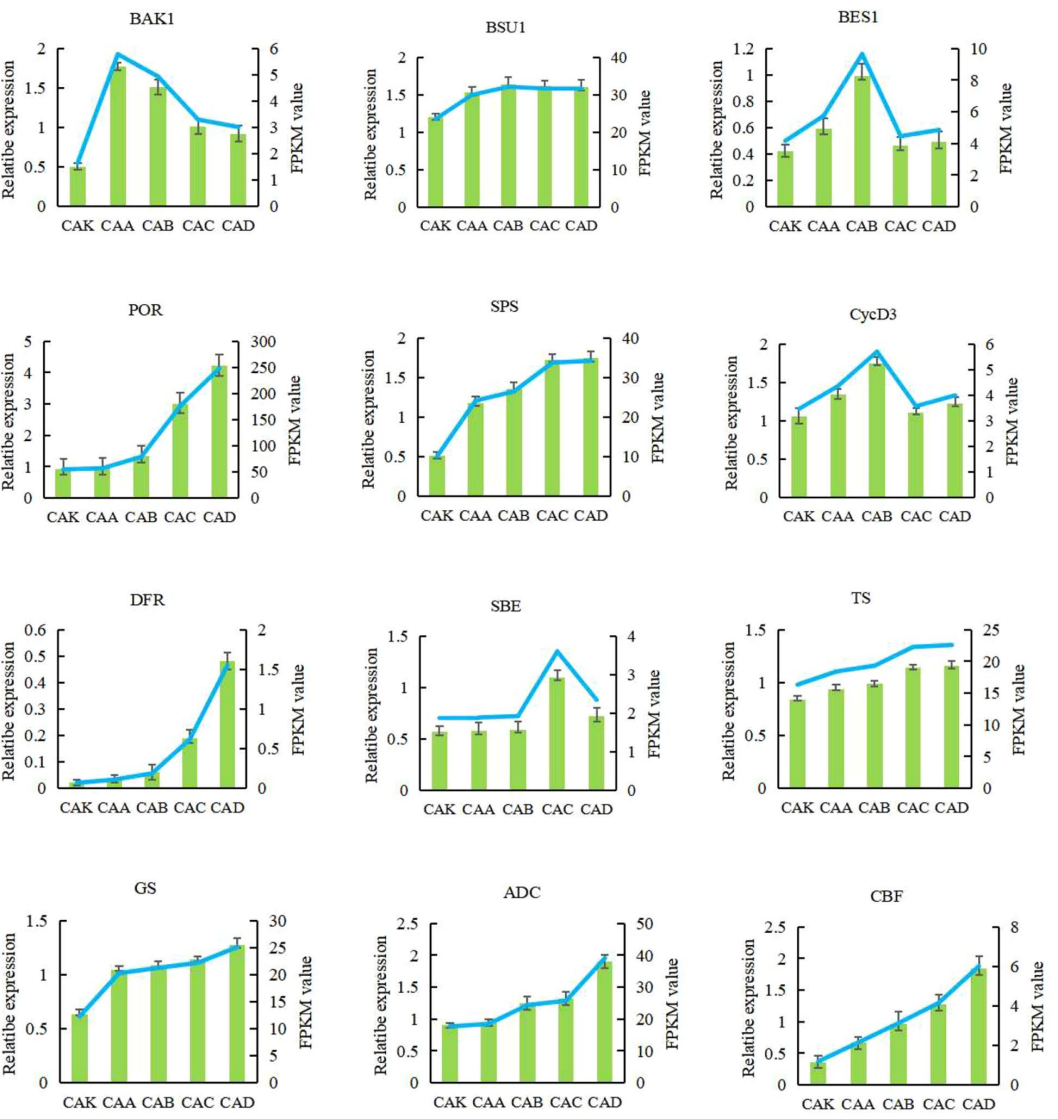
Twelve candidate genes were verified and measured by real-time fluorescence quantitative PCR. The data represent the average ± standard deviation (*n* = 3). The vertical axis represents the level of gene expression found through PCR. There was a significant difference between tea leaves treated with brassinosteroids (BRs) for 0 h and tea leaves exogenously sprayed with BRs for 48 h (*P* < 0.05)

To confirm the gene expression patterns detected on the transcriptome dataset, qRT-PCR analysis was performed to determine the mRNA expression of BAK1, BES1, BSU1, SPS, SBE, protochlorophyllide oxidoreductase (POR), DFR, CycD3, threonine synthase (TS), glutamine synthetase (GS), arginine decarboxylase (ACD), and inducer of C-repeat-binding factor expression (ICE) in the five samples (Fig. 3). The expression profiles of the single genes detected in qRT-PCR analysis coincided with those detected in the RNA-seq datasets.

### Exogenous spraying of BR onto tea leaves promotes the upregulated expression of genes involved in the BR signal transduction pathway

**Fig. 4:**
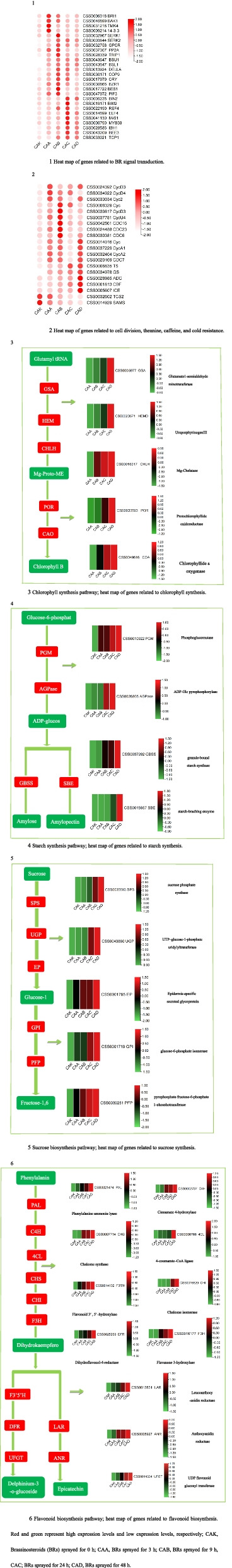
1 Heat map of genes related to BR signal transduction. 2 Heat map of genes related to cell division, theanine, caffeine, and cold resistance. 3 Chlorophyll synthesis pathway; heat map of genes related to chlorophyll synthesis. 4 Starch synthesis pathway; heat map of genes related to starch synthesis. 5 Sucrose biosynthesis pathway; heat map of genes related to sucrose synthesis. 6 Flavonoid biosynthesis pathway; heat map of genes related to flavonoid biosynthesis. Red and green represent high expression levels and low expression levels, respectively; CAK, Brassinosteroids (BRs) sprayed for 0 h; CAA, BRs sprayed for 3 h; CAB, BRs sprayed for 9 h, CAC; BRs sprayed for 24 h; CAD, BRs sprayed for 48 h

KEGG enrichment annotation revealed that 26 genes are involved in the BR signal transduction pathway (Fig. 4: 1). KEGG analysis showed that compared with CAK (BR spraying for 0 h), the expression levels of BRI1, BAK1, transmembrane kinase 4 (TMK4), 14-3-3, abscisic acid G-protein coupled receptor (GPCR), BSU1, BES1, and BES1-interacting myc-like 2 (BIM2) that are related to BR signal transduction were upregulated after BR spraying (for 3 h, 9 h, 24 h, and 48 h), but the highest gene expression levels varied among time points, which may be due to the different sequences of signal transduction.

### Exogenous spraying of BR promotes cell division, theanine synthesis, and increased expression of genes related to cold resistance in tea leaves

KEGG enrichment and annotation revealed that numerous cyclin genes in tea leaves were upregulated by BR spraying (Fig. 4: 2). In addition, three genes for theanine synthesis and one gene related to cold resistance were also identified (Fig. 4: 2). KEGG analysis showed that compared with CAK (BR spraying for 0 h), the expression levels of several mitotic cyclin genes such as *Cyc*, *CycD3*, *CycD4*, and *CDC6* were upregulated 3 h, 9 h, 24 h, and 48 h after BR spraying, but the highest gene expression levels varied among time points. We hypothesize that within 48 h of BR spraying, cyclin genes were upregulated, which in turn promoted growth through cell division. In addition, it was found that spraying BRs onto tea leaves also significantly upregulated the cold resistance genes *CBF* and *ICE*, as well as the theanine synthesis-related genes threonine synthase, (TS), glutamine synthetase (GS), and arginine decarboxylase (ADC). Interestingly, the expression of caffeine-related synthetic genes was downregulated such as caffeine synthase 2(TCS2) and S-adenosylmethionine synthase (SAMS).

### Exogenous spraying of BR upregulates genes related to the chlorophyll biosynthetic pathway in tea leaves

KEGG enrichment annotation identified five genes in the ginseng chlorophyll biosynthesis pathway (Fig. 4: 3). KEGG analysis showed that compared with CAK (BR spraying for 0 h), after BR spraying for 3 h, 9 h, 24 h, and 48 h, the key regulatory genes of glutamate-1-semialdehyde aminotransferase (GSA), uroporphyrinogen III synthase (HEMD), POR, Mg-chelatase (C-HLH), and chlorophyllide a oxygenase (CAO) that are related to chlorophyll synthesis pathway were upregulated, and their expression levels peaked at 48 h.

### Exogenous spraying of BR onto tea leaves promotes the upregulated expression of genes related to the starch biosynthesis pathway

KEGG enrichment annotation revealed that three genes are involved in the starch biosynthesis pathway (Fig. 4: 4). KEGG analysis showed that compared with CAK (spraying BRs for 0 h), after spraying BRs for 3 h, 9 h, 24 h, and 48 h, the expression of ADP-Glc pyrophosphorylase (AGPase), GBSS, phosphoglucomutase (PGM), and the starch-branching enzyme (SBE) key regulatory bases related to the starch synthesis pathway were up-regulated. At 24 h, the expression of genes related to the sucrose synthesis pathway peaked.

### Exogenous spraying of BR onto tea leaves promotes the upregulated expression of genes in the sucrose biosynthetic pathway

Eight genes involved in the sucrose biosynthesis pathway were identified by KEGG enrichment annotation (Fig. 4: 5). KEGG analysis showed that compared with CAK (BR spraying for 0 h), the expression of the UTP-glucose-1-phosphate uridylyltransferase (UGP), *SPS*, glucose-6-phosphate isomerase (GPI), pyrophosphate fructose-6-phosphate 1-phosphotransferase (PFP), and epidermis-specific secreted glycoprotein (EP) key regulatory genes related to the sucrose biosynthesis pathway were upregulated after BR spraying for 3 h, 9 h, 24 h, and 48 h.

### Exogenous spraying of BR onto tea leaves promotes the upregulated expression of genes in the biosynthetic pathway of flavonoids

Eleven genes involved in flavonoid biosynthesis were identified by KEGG enrichment annotation (Fig. 4: 6). The flavonoid biosynthesis-related genes PAL, C4H, 4CL, chalcone synthase (CHS), chalcone isomerase (CHI), flavanone 3-hydroxylase (F3H), flavonoid 3′,5′-hydroxylase (F3’5’H), DFR, LAR, ANR, and UFGT were upregulated, with peak values observed at 48 h.

## Discussion

### BR signal transduction mechanism in tea leaves

**Fig. 5:**
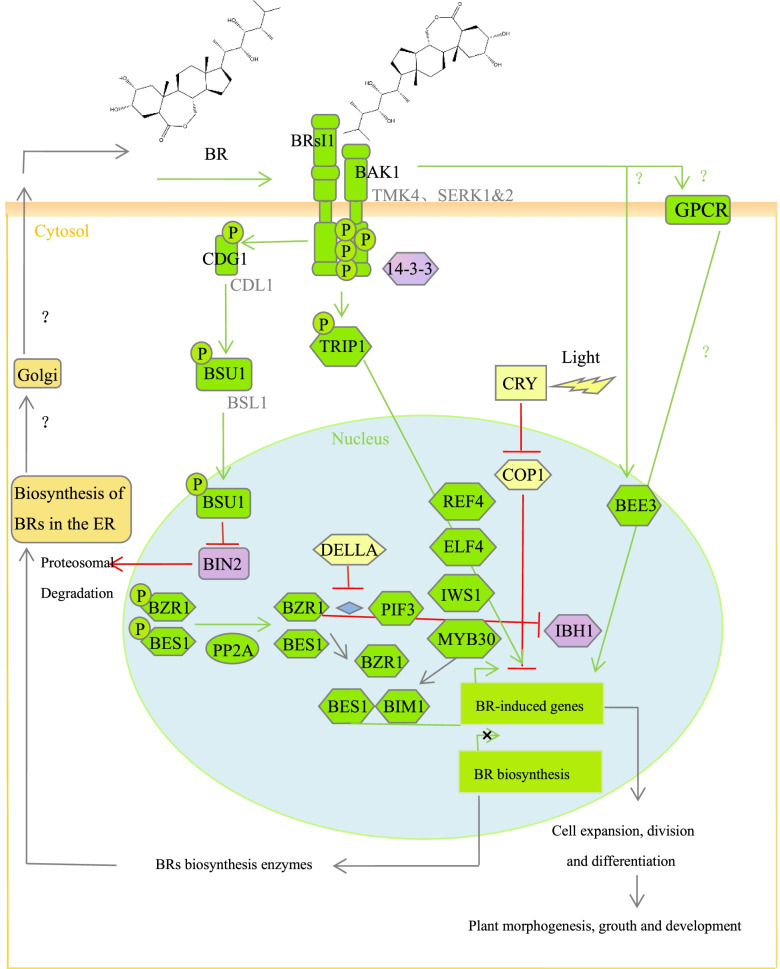
A possible model of the BR signaling pathway with BRs (the activation state of BR signaling) sprayed onto tea leaves

Through KEGG enrichment and annotation, 26 genes involved in the BR signal transduction pathway were identified. According to the heat maps of genes related to BR signal transduction under different BR treatments, it was found that 26 genes in the BR signal transduction pathway were significantly upregulated with increasing BR spraying time. Combined with the BR signal transduction maps of Arabidopsis and rice, we describe a possible model of the BR signal pathway in tea leaves [29–41] (Fig. 5). At present, the signal transduction pathway of BR in Arabidopsis and rice has been reported. Compared with rice, the signal transduction pathway of BR in tea leaves is similar to that of Arabidopsis [24]. Unlike the BR signal transduction pathway in *A. thaliana*, BAK1-like kinase contains both SERK and TMK4 in the BR signal transduction pathway of tea leaves. In our transcriptome data, the ATBS1-interacting factors (AIF) and paclobutrazol resistance 1 (PRE) genes did not significantly differ in expression levels, whereas that of the teosinte branched (TCP) gene was significant. AIF is the negative regulator of BR signal transduction, while PRE and TCP are the positive regulators of BR signal transduction [34]. The results showed that TCP, the forward regulator of BR signal transduction, plays a leading role in the effects of the exogenous spraying of BRs onto young tea leaves.

### Exogenous spraying of BR promotes the growth and development of tea plants

Through KEGG enrichment and annotation, the *UGP*, *SPS*, *GPI*, *PFP* and *EP* genes involved in sucrose synthesis; the *GSA*, *HEMD*, *POR*, *CHLH*, and *COA* genes related to ginseng chlorophyll synthesis; the *AGPase*, *GBSS*, and *SBE* genes related to starch synthesis; and the flavonoid biosynthesis-related *PAL*, *C4H*, *4CL*, *CHS*, *CHI*, *F3H*, *F3’5′*, *DFR*, *LAR*, *ANR*, and *UFGT* genes were identified. The results showed that exogenous spraying of BRs upregulated the expression of genes related to sucrose synthesis, chlorophyll synthesis, starch synthesis, and flavonoid biosynthesis. It can be inferred that exogenous BR spraying increased the content of sucrose, chlorophyll, starch, and flavonoids. In addition, a large number of highly expressed cyclin genes, including *Cyc*, *CycD3*, *CycD4*, and *CDC6*, were found. Cell cycle regulatory proteins can bind to cell differentiation cycle-coding proteins and activate corresponding protein kinases, thus promoting cell division. BRs can enhance plant growth by promoting cell division. The above conclusions are consistent with the research findings on *Arabidopsis thaliana* and rice. We also found that exogenous BR spraying upregulates theanine synthesis genes, namely, *TS*, *GS*, and *ADC* and cold resistance-related genes, namely, *CBF*, *ICE*. It can be inferred that exogenous BR spraying increased the theanine content in tea leaves and improved cold resistance of tea plants. Theanine (L-Theanine) is a unique free amino acid in tea and main component of tea. Our findings are concordant to the research results of Li et al. (2018).

The effect of exogenous BR spraying on the growth and development of tea leaves and the signal transduction pathway of BR in tea leaves was revealed by transcriptome analysis. Our results showed that the tea leaves sprayed with BRs were significantly different, and the upregulated genes were mainly related to BR signal transduction, sucrose synthesis, chlorophyll synthesis, starch synthesis, flavonoid biosynthesis, cell division, theanine synthesis, and cold resistance. In addition, we also found that after spraying BR, the key genes for caffeine synthesis were down-regulated. Our study lays the foundation for elucidating the molecular mechanism of the BR signal transduction pathway in tea leaves and its regulatory role on the growth and development of tea plants.

## Supplementary Information

**Additional file 1.**

## Data Availability

All the data supporting our findings are contained within the manuscript. All raw transcriptome data reported in this article have been deposited in the Sequence Read Archive (SRA) under accession number PRJNA756445.
